# Association between balance impairment and incidence of motoric cognitive risk syndrome in the China Health and Retirement Longitudinal Study

**DOI:** 10.1016/j.jnha.2024.100476

**Published:** 2025-01-08

**Authors:** Zhigang Xu, Shuli Jia, Ning Huang, Ya Ma, Dan Qin, Birong Dong

**Affiliations:** a The Center of Gerontology and Geriatrics and National Clinical Research Center of Geriatrics, West China Hospital, Sichuan University, China; b The Affiliated Traditional Chinese Medicine Hospital, Southwest Medical University, China; c The People’s Hospital of Yubei District of Chongqing, China

**Keywords:** Motoric cognitive risk syndrome, Balance, Cognitive function, Older adults

## Abstract

**Objectives:**

Motor cognitive risk (MCR) syndrome, defined as the cooccurrence of subjective cognitive complaints and a slow gait speed, is a form of pre-dementia condition. Balance has previously been associated with cognitive function. However, to date, no study has examined the relationship between balance and MCR in a large cohort of older adults. We aimed to investigate the associations of balance with MCR among Chinese older adults.

**Research design and methods:**

Data from the wave 1 to wave 3 of the China Health and Retirement Longitudinal Study (CHARLS) were used. Balance was measured using validated tandem stance. Logistic and discrete-time survival cox regression analyses were performed to examine the relationship between baseline balance impairment and prevalent and incident MCR.

**Results:**

A total of 3,398 participants were included in the baseline study. The prevalence of balance impairment was 21.1%. In the cross-sectional analysis, balance impairment was significantly associated with higher odds of MCR in the fully-adjusted model (OR: 1.43 95%CI 1.14–1.80, p = 0.002). A total of 2,474 individuals were included in the longitudinal analysis. During a mean follow-up duration of 3.69 years, the incidence of MCR was 9.8%. Baseline balance impairment was also significantly related to incidence of MCR (HR:1.37 95%CI 1.03–1.82, p = 0.032) even adjusting all confounders.

**Conclusion:**

These results show that early recognition of balance disorder may be helpful in the prevention and treatment of cognitive decline in older adults.

## 1 Introduction

As the global population ages, the incidence of age-related diseases is increasing each year. Cognitive impairment is a pressing public health issue that requires urgent attention. Data indicate that over 50 million individuals worldwide are affected by Alzheimer's disease and related dementias (ADRD), and this number is expected to rise in conjunction with the aging population [1]. Due to the irreversible nature of cognitive impairments and the limitations of current effective interventions, there is a strong emphasis on early detection and early intervention. Identifying high-risk individuals for cognitive impairment in advance can facilitate early information acquisition and enable earlier intervention.

Motoric cognitive risk syndrome (MCR) is a pre-dementia disease, defined as the co-occurrence of subjective cognitive complaints and a slow gait speed [2]. MCR is associated with higher risk of Alzheimer's disease (AD) and vascular dementia, and is concurrently accompanied by falls and movement abnormalities [2,3]. The investigation of factors associated with MCR serves as an excellent entry point for early interventions in dementia. Identifying risk factors for MCR earlier for intervention or prevention may represent a more strategic approach to reducing the incidence of MCR and cognitive impairment. Balance relies on signals and feedback from the vestibular system and brain, providing a physiological link between the two [[4], [5], [6]]. The mobility of older adults is compromised prior to the decline in cognitive abilities [7]. Research has found that a decline in balance ability occurs more frequently in dementia, significantly exceeding the measurements of physical function [8]. It is possible that balance ability serves as a precursor to the decline in cognitive function during the aging process. Determining the potential of balance as a risk factor for MCR has the possibility to inform interventions that prevent cognitive decline in older population in the future.

Currently, there is no research examining the relationship between balance and MCR based on a large sample with prospective study design. The objective of this study was to evaluate the cross-sectional and longitudinal relationship between standing balance and MCR in a cohort of Chinese older adults. We hypothesised that worse balance ability would be associated with higher risk of MCR.

## 2 Methods

### 2.1 Study population

The data of the present study were from the China Health and Retirement Longitudinal Study (CHARLS), a longitudinal study designed to examine health and economic adjustments in response to the rapidly aging population in China [9]. The baseline survey for the study began in 2011 (Wave 1), including 17708 respondents aged 45 years and older. Participants were subsequently followed up every two years, with four additional waves conducted in 2013 (Wave 2), 2015 (Wave 3), 2018 (Wave 4), and 2020 (Wave 5). Further information about CHARLS can be found in other resources [9].

**Fig. 1:**
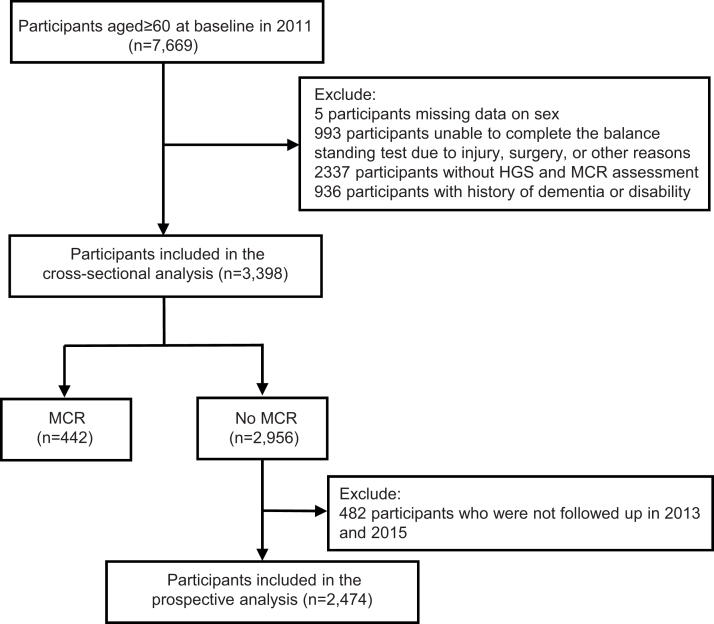
Study flow.

We used three waves (wave 1 as baseline data, wave 2 and wave 3 as follow up data) of CHARLS to investigate the association of balance impairment with the incidence of MCR. The population selection flow was present in Fig. 1. We included an initial sample of 3,998 participants aged ≥60 years at baseline in 2011. Balance was assessed at baseline, while subjective cognitive complaints and gait speed were evaluated at baseline, 2013 and 2015 follow-up surveys. Participants with a history of dementia or disability at baseline were excluded from the study. Additionally, individuals who were classified as MCR at baseline, those who were not successfully followed up, and those who did not complete the MCR assessment in 2013 and 2015 were also excluded from the longitudinal analyses. As a result, the final sample for the prospective analyses comprised 2,474 participants.

The CHARLS protocol was approved by the Institutional Review Board of Peking University (No. IRB00001052-11015) and the written informed consent was obtained from all participants. The data used in the current analysis are publicly available through the CHARLS website (http://charls.pku.edu.cn/).

### 2.2 Evaluation of balance

Balance measures were assessed using validated tandem stance measures. Normal balance was defined as full-tandem stance, which required participants to stand with one foot in front of the other, for 30 s (≥70 years), and 60 s (<70 years); otherwise, balance is considered impaired [10].

### 2.3 Assessment of MCR

MCR assessment was based on the method proposed by Verghese et al. [11], defined as subjective cognitive complaints (SCC) and slow gait speed (SG) but without dementia or mobility disability. Participants with self-reported physical disability or mobility disability defined as “needing assistance” when performing activities of daily living. SCC were measured based on a “fair” or “poor” response to the following question “How would you rate your memory at the present time?”. Lastly, infrared sensors were used to measure the speed of usual-pace walking over a 2.5-m distance. SG was defined by gait speed greater than or equal to 1 SD below the average of age- and sex-specific values. The cut-off values of slow gait for people <75 and ≥75 years old were 0.50 m/s, 0.40 m/s in men, and 0.47 m/s, 0.40 m/s in women, which are similar to those in other studies [12].

### 2.4 Covariates

To address potential confounding factors, we adjusted for various sociodemographic, lifestyles, and health characteristics at baseline. These included age, sex (male/female), residence (urban/rural), marital status (married/widowed/other), education level (illiterate/uncompleted primary school/primary school/middle school/high school and above), smoking status (never/former/current smoker), drinking status (never/former/current drinker), body mass index (BMI), and medical history regarding diabetes, heart disease, hypertension, lung diseases, asthma, stroke, arthritis, and dyslipidemia (yes/no).

### 2.5 Statistical analysis

T-test and Chi-square test were used to test the differences of the baseline sociodemographic, lifestyle and health characteristics between the 2 groups. Univariate and multivariate logistic regression were performed to calculate odds ratios (ORs) and 95% confidence intervals (95% CIs) of association between baseline balance impairment status and MCR in cross-sectional analyses. After excluding prevalent MCR in baseline, we conducted the discrete-time survival cox regression (DTSA) to examine the association between the baseline balance status and MCR incidence during the follow-up. The covariates adjusted in the multivariate regression model included age, sex, race, residence, marital status, education, smoking status, drinking status, BMI, and history of chronic diseases at baseline. All statistical analyses were performed using 15.1 software (Stata Corp, College Station, TX), and P < 0.05 (two-tailed) indicated statistical significance.

## 3 Results

### 3.1 Baseline characteristics of study population

A total of 3,398 participants were included in the baseline study. The baseline characteristics of participants grouped by balance impairment status are present in Table 1. The prevalence of balance impairment was 21.1% (844/3398). The prevalence of MCR was 13%. Among the total participants, the mean age was 67.0. Compared with participants with normal balance, those with balance impairment were more likely to be females, widowed, and lower educational levels; they were older and had higher prevalence of heart diseases, hypertension, stroke, arthritis and higher BMI. They also had higher prevalence of MCR.

**Table 1:** Participant Characteristics by balance status at baseline in the China Health and Retirement Longitudinal Study, 2011 (n = 3,398).

Characteristic	Overall n = 3,398	Normal balance n = 2,554	Balance impairment n = 844	P value
Age, mean ± SD	67.0 ± 5.9	66.7 ± 5.7	67.8 ± 6.4	<0.001
Sex, n (%)				<0.001
Male	1,885 (55.5)	1,530 (59.9)	355 (42.1)	
Female	1,513 (44.5)	1,024 (40.1)	489 (57.9)	
Residence, n (%)				0.43
Rural	2,107 (62.0)	1,574 (61.6)	533 (63.2)	
Urban	1,291 (38.0)	980 (38.4)	311 (36.8)	
Marital Status, n (%)				<0.001
Married	2,720 (80.0)	2,080 (81.4)	640 (75.8)	
Widowed	521 (15.3)	346 (13.5)	175 (20.7)	
Others	157 (4.6)	128 (5.0)	29 (3.4)	
Education, n (%)				<0.001
Illiteracy	930 (27.4)	629 (24.6)	301 (35.7)	
Unfinished primary school	724 (21.3)	557 (21.8)	167 (19.8)	
Primary school	1,022 (30.1)	782 (30.6)	240 (28.4)	
Middle school	485 (14.3)	386 (15.1)	99 (11.7)	
High school and above	236 (6.9)	199 (7.8)	37 (4.4)	
Smoke, n (%)				<0.001
Never smoke	1,808 (53.2)	1,301 (51.0)	506 (60.0)	
Former smoke	418 (12.3)	333 (13.0)	85 (10.1)	
Current smoke	1,171 (34.5)	919 (36.0)	252 (29.9)	
Drink, n (%)				0.001
Never drink	1,864 (54.9)	1,359 (53.3)	505 (59.8)	
Former drink	1,387 (11.4)	289 (11.3)	98 (11.6)	
Current drink	1,143 (33.7)	902 (35.4)	241 (28.6)	
BMI, mean ± SD	22.8 ± 3.4	22.7 ± 3.4	23.1 ± 3.6	0.005
History of diabetes, n (%)	209 (6.2)	147 (5.8)	62 (7.4)	0.093
History of heart disease, n (%)	482 (14.3)	336 (13.2)	146 (17.4)	0.003
History of hypertension, n (%)	993 (29.3)	707 (27.8)	286 (33.9)	<0.001
History of chronic lung diseases, n (%)	439 (13.0)	337 (13.2)	102 (12.1)	0.39
History of asthma, n (%)	174 (5.1)	126 (4.9)	48 (5.7)	0.38
History of stroke, n (%)	69 (2.0)	43 (1.7)	26 (3.1)	0.012
History of dyslipidemia, n (%)	348 (10.4)	251 (9.9)	97 (11.7)	0.14
History of arthritis, n (%)	1,188 (35.0)	869 (34.1)	319 (37.9)	0.045
MCR, n (%)	442 (13.0)	300 (11.7)	142 (16.8)	<0.001

BMI, body mass index; MCR, motoric cognitive risk syndrome.

### 3.2 Association between balance and MCR

Table 2 shows the cross-sectional association between baseline balance impairment status and prevalence of MCR. Balance impairment was significantly associated with higher odds of MCR in the unadjusted model (OR: 1.52 95%CI 1.22–1.89, p < 0.001). The results remain significant when adjusting age, sex, residence, marital status, education, smoking status, drinking status, BMI and chronic diseases (OR: 1.43 95%CI 1.14–1.80, p = 0.002). A total of 2,474 participants were included in the longitudinal analysis, with a mean follow-up duration of 3.69 years. During this period, 243 cases of MCR were identified. The longitudinal associations between baseline balance impairment status and incidence of MCR were present in Table 3. In the unadjusted model, compared with normal balance in baseline, participants with balance impairment were significantly associated with increased risk of incidence of MCR (HR: 1.67 95%CI 1.27–2.18, p < 0.001). In the fully adjusted model, baseline balance impairment was also significantly related to MCR (HR:1.37 95%CI 1.03–1.82, p = 0.032).

**Table 2:** Cross-sectional associations of Balance Status and MCR in the China Health and Retirement Longitudinal Study (n = 3398).

Balance status	Unadjusted model		Adjusted model^a^	
	OR (95%CI)	P value	OR (95%CI)	P value
Normal balance	1 (Reference)	NA	1 (Reference)	NA
Balance impairment	1.52 (1.22,1.89)	<0.001	1.43 (1.14,1.80)	0.002

MCR, motoric cognitive risk syndrome; OR: odds ratio; CI: confidence interval.

a Adjusted for age, sex, residence, marital status, education, smoking status, drinking status, BMI, and chronic diseases.

**Table 3:** Prospective associations of baseline balance status and 4-year incidence of MCR in the China Health and Retirement Longitudinal Study (n = 2474).

	Unadjusted model		Adjusted model^a^	
Balance status	HR (95%CI)	P value	HR (95%CI)	P value
Normal balance	1 (Reference)	NA	1 (Reference)	NA
Balance impairment	1.67 (1.27,2.18)	<0.001	1.37 (1.03,1.82)	0.032

MCR, motoric cognitive risk syndrome; HR: Hazard ratio; CI: confidence interval.

a Adjusted for age, sex, residence, marital status, education, smoking status, drinking status, BMI, and chronic diseases.

## 4 Discussion

In this cohort of Chinese older adults, we examined the relationship between balance impairment and prevalence and incidence of MCR. The main results suggest that balance impairment was significantly associated with prevalence and incidence of MCR, which indicates routine balance screenings in the older adults can help identify at-risk individuals early and facilitate balance training, thereby delaying or preventing further decline in cognitive function.

### 4.1 Main findings

In this study, the prevalence of MCR was found to be 13%. Previous studies conducted in France report a prevalence of approximately 9.0% [13], while the prevalence in the United States is 7.0% and in Japan is 6.3% [14]. Notably, a related study from Mexico indicates a prevalence of 14.3% [15], which is similar to the findings of this study. Additionally, a study from France reports a prevalence of 14.23% [16]. Conversely, the lowest prevalence of MCR is observed in Ireland, which stands at 2.56% [17]. MCR is associated with age, educational attainment, body mass index (BMI), depression, pain, frailty, cognitive scores, and grip strength [[18], [19], [20], [21]]. Additionally, nutritional intake (such as vitamin K) [22], cardiovascular disease risk factors (such as smoking and hypercholesterolemia) [23], as well as stroke [24] and transient ischemic attacks [25] are also correlated with MCR.

We found that balance impairment is associated higher odds of MCR based on cross-sectional and longitudinal study. Age- and disease-related changes in the central and peripheral nervous system result in balance impairment in older adults [26]. In this study, the prevalence of balance disorders was found to be 21.1%. Compared to participants with normal balance abilities, those with balance disorders were more likely to be female, widowed, and have a lower level of education. Furthermore, the prevalence of cardiovascular disease, hypertension, stroke, and arthritis among patients with balance disorders in this study is significantly higher, along with an elevated body mass index. Coronary atherosclerotic heart disease, hypertension, stroke, and arthritis play significant roles in the occurrence and progression of MCR [15,27,28]. Research has reported that the prevalence of MCR in elderly women doubles every decade, whereas this trend is not observed in men, which aligns with the higher incidence of balance disorders among women in this study [29].

Our research findings indicate that the presence of balance disorders may exacerbate the decline in cognitive abilities. Balance relies on signals and feedback from the vestibular system and brain, providing a physiological link between the two [[4], [5], [6]]. Decline in vestibular function is associated with a decrease in cognitive abilities, which may also be one of the reasons for cognitive decline resulting from balance disorders [[30], [31], [32]]. Age-related changes in sensory systems, including alterations in vision, vestibular function, and proprioception, can lead to balance issues and a decline in motor function [33]. As individuals age, the reduction in muscle strength increases the risk of falls, balance disorders, and movement impairments [34,35], with a more pronounced effect observed in females [36]. The subcortical circuits of the frontal lobe are crucial components in regulating gait activities and cognitive functions, primarily overseeing attention and executive functions. They play a significant role in the coordination of motor, sensory, and cognitive processes [37]. When these structures are subjected to focal or diffuse damage, it may adversely affect balance and cognitive capabilities.

### 4.2 Implications

Our study has important clinical and public health implications. First, integrating balance assessment into routine geriatric assessment in older adults is necessary. Individuals with balance impairment should be considered as the primary target to prevent MCR and cognitive decline. Normal balance individuals also need to evaluate the risk factors of balance impairment so that at-risk individuals can be identified early and the corresponding prevention measures can be performed to delay the cognitive impairment progression. Furthermore, balance impairment is also a reversible state. Therefore, many efforts are needed to conduct effective interventions for balance training.

### 4.3 Advantages and limitations

Our study presents several advantages. First, to the best of our knowledge, this is the first study to investigate the associations between balance and risk of prevalence and incidence of MCR. Furthermore, prospective study design and large sample sizes enhances the reliability of the results. Our study also has limitations. First, selection bias might occur in the present study because we excluded participants who did not complete balance tests. Second, although we adjusted for multiple covariates, residual confounding might remain. At last, the participants were from community, further studies are needed to conformed our conclusions in clinical patients.

## 5 Conclusion

Our results provide evidence for balance impairment and MCR, in a community-dwelling older adults. The results indicate that balance impairment was associated higher risk of MCR. Routine screening balance ability may be beneficial to early identify participants who were at higher risk of cognitive decline and facilitate early interventions to delay or prevent cognitive deterioration.

## CRediT authorship contribution statement

Conceptualization: Zhigang Xu, Shuli Jia, Birong Dong; Data curation: Shuli Jia; Formal analysis: Shuli Jia; Funding acquisition: Ning Huang, Birong Dong; Methodology: Zhigang Xu, Shu Jia; Visualization: Shuli Jia; Writing – original draft: Zhigang Xu; Writing – review and editing: Shuli Jia, Ning Huang, Birong Dong

## Ethics approval and consent to participate

The CHARLS protocol was approved by the Institutional Review Board of Peking University (No. IRB00001052-11015) and the written informed consent was obtained from all participants.

## Study funding

This work was partially supported by the National Clinical Research Center for Geriatrics, West China Hospital, Sichuan University (Y2022JC004); 1.3.5 project for disciplines of excellence, West China Hospital, Sichuan University (ZYGD20010); Sichuan Science and Technology Program (No. 2023NSFSC1158); Project funded by China Postdoctoral Science Foundation (No. 2023M732473); National Clinical Research Center for Geriatrics, West China Hospital (No. Z2024JC006); These study fundings had no role in study design, collection, analysis and interpretation of data, writing of the report and decision to submit the article for publication.

## Declaration of competing interest

We, the authors, declare that there are no conflicts of interest regarding the publication of this paper. We confirm that the work described in this manuscript has not been published before, and it is not under consideration for publication elsewhere. We also confirm that all authors have read and approved the manuscript and that there are no known conflicts of interest associated with this publication.

## Glossary

CHARLS: China Health and Retirement Longitudinal Study
MCR: motoric cognitive risk
OR: odds ratio
HR: Hazards Ratio
KFACS: The Korean frailty and aging cohort study
BMI: body mass index
